# Transcranial electrostimulation with special waveforms enhances upper-limb motor function in patients with chronic stroke: a pilot randomized controlled trial

**DOI:** 10.1186/s12984-021-00901-8

**Published:** 2021-06-30

**Authors:** Shih-Ching Chen, Ling-Yu Yang, Muhammad Adeel, Chien-Hung Lai, Chih-Wei Peng

**Affiliations:** 1 Department of Physical Medicine and Rehabilitation, School of Medicine, College of Medicine, Taipei Medical University, Taipei, Taiwan; 2 Department of Physical Medicine and Rehabilitation, Taipei Medical University Hospital, Taipei, Taiwan; 3 School of Biomedical Engineering, College of Biomedical Engineering, Taipei Medical University, Taipei, Taiwan; 4 International PhD Program in Biomedical Engineering, College of Biomedical Engineering, Taipei Medical University, Taipei, Taiwan; 5 School of Gerontology Health Management, College of Nursing, Taipei Medical University, 250 Wuxing Street, Taipei, 11031 Taiwan

**Keywords:** Stroke, Transcranial direct current stimulation (tDCS), Intermittent theta burst stimulation (iTBS), Upper limb, Functional recovery

## Abstract

**Background:**

Transcranial direct current stimulation (tDCS) and intermittent theta burst stimulation (iTBS) were both demonstrated to have therapeutic potentials to rapidly induce neuroplastic effects in various rehabilitation training regimens. Recently, we developed a novel transcranial electrostimulation device that can flexibly output an electrical current with combined tDCS and iTBS waveforms. However, limited studies have determined the therapeutic effects of this special waveform combination on clinical rehabilitation. Herein, we investigated brain stimulation effects of tDCS-iTBS on upper-limb motor function in chronic stroke patients.

**Methods:**

Twenty-four subjects with a chronic stroke were randomly assigned to a real non-invasive brain stimulation (NIBS; who received the real tDCS + iTBS output) group or a sham NIBS (who received sham tDCS + iTBS output) group. All subjects underwent 18 treatment sessions of 1 h of a conventional rehabilitation program (3 days a week for 6 weeks), where a 20-min NIBS intervention was simultaneously applied during conventional rehabilitation. Outcome measures were assessed before and immediately after the intervention period: Fugl-Meyer Assessment-Upper Extremity (FMA-UE), Jebsen-Taylor Hand Function Test (JTT), and Finger-to-Nose Test (FNT).

**Results:**

Both groups showed improvements in FMA-UE, JTT, and FNT scores after the 6-week rehabilitation program. Notably, the real NIBS group had greater improvements in the JTT (*p* = 0. 016) and FNT (*p* = 0. 037) scores than the sham NIBS group, as determined by the Mann–Whitney rank-sum test.

**Conclusions:**

Patients who underwent the combined ipsilesional tDCS-iTBS stimulation with conventional rehabilitation exhibited greater impacts than did patients who underwent sham stimulation-conventional rehabilitation in statistically significant clinical responses of the total JTT time and FNT after the stroke. Preliminary results of upper-limb functional recovery suggest that tDCS-iTBS combined with a conventional rehabilitation intervention may be a promising strategy to enhance therapeutic benefits in future clinical settings.

*Trial registration:* ClinicalTrials.gov Identifier: NCT04369235. Registered on 30 April 2020.

## Introduction

Neuromodulation is an evolving therapy for rehabilitation after a stroke and is also used to improve motor function in the lesioned cortex. Recently, studies indicated that neuromodulation could enhance neuroplasticity, the ability of the brain to reorganize or relearn in response to a new stimulus, resulting in facilitation of motor sensory recovery in stroke patients [1–3]. Transcranial direct current stimulation (tDCS), a non-invasive brain stimulation (NIBS) technique, is contemporarily important as it can modulate neuroplasticity in advanced rehabilitation medicine, such as pain, depression and, addictive diseases [4–6]. tDCS can selectively change the excitability of the regional cortex non-invasively and safely [7]. In addition, tDCS has been explored as a treatment option for stroke, particularly for upper/lower-limb motor function [8–11]. However, studies reported only 10% ~ 30% improvement in forearm motor function after stroke rehabilitation. Optimal stimulation strategies of tDCS to improve plasticity and enhance motor learning need to be determined.

Recovery as a result of traditional stroke rehabilitation often has poor outcomes and long rehabilitation times. Therefore, developing a more-effective therapeutic device is an important issue for stroke rehabilitation. To develop an optimal tDCS protocol to improve motor function, we designed and implemented a prototype of a novel transcranial electrostimulation device that can flexibly output an electrical current waveform by combining DC and theta burst waveforms [12]. Theta burst stimulation (TBS) was originally a novel waveform of repetitive transcranial magnetic stimulation (rTMS) that is more rapid and efficacious than rTMS [13]. Numerous studies determined that TBS has more advantages than other traditional waveforms of rTMS, such as long-lasting effects on motor-evoked potentials (MEPs) and neuronal excitability after a shorter stimulation duration [14–16], and it was associated with fewer adverse events [17]. It is well known that the most widely used TBS patterns are intermittent (i)TBS and continuous (c)TBS. iTBS consists of a 2-s train of TBS repeated every 10 s for a total of 190 s which produces long-term potentiation (LTP)-like effects, whereas cTBS consists of three-pulse bursts at 50 Hz repeated every 200 ms for 40 s, which induces long-term depression (LTD)-like cortical plasticity [14, 18–20].

Use of an rTMS protocol with iTBS in chronic stroke patients was shown to significantly increase ipsilesional M_1_ excitability, enhanced MEP amplitudes, and improve upper-limb motor functions [15, 21–23]. One recent meta-analysis showed that the standardized mean difference (SMD) of iTBS was 0.60 (*p* = 0.018), whereas that for cTBS was 0.35 (*p* = 0.138) for the recovery of upper-limb motor outcomes in stroke patients, indicating that iTBS was more beneficial than cTBS in motor recovery after a stroke [24]. Therefore, modulation of cortical plasticity induced by iTBS may have therapeutic potential for patients with post-stroke motor disorders.

Both rTMS and tDCS can cause physiological effects and indirectly modulate deep-brain locations via neural circuits [25, 26]. In general, rTMS therapy is usually applied before undertaking occupational therapy for patients with motor function deficits, due to the bulky size of the rTMS device. On the contrary, the lightweight, portable tDCS device can be directly worn on a patient's head during active rehabilitation exercises, which was associated with augmentation of synaptic plasticity [27–29]. However, most traditional transcranial stimulators have only a DC waveform mode at present. Thus, our novel transcranial burst electrostimulator was designed to develop an effective and optimal therapeutic system for patients who need rehabilitation therapy. We previously demonstrated that compared to conventional anodal tDCS, the combined DC-iTBS electrostimulator induced LTP-like plasticity as evident from significantly enhanced MEP amplitudes for at least 30 min in animal experiments [12].

With the excellent efficacy of previously combined stimulation, we report a pilot randomized controlled study to examine the combined effects of DC-iTBS and conventional rehabilitation (CR) on upper-limb motor function as measured by the Fugl-Meyer Assessment upper extremity (FMA-UE), Finger-to-Nose test (FNT), and Jebsen-Taylor hand function test (JTT) in patients with chronic stroke compared to a sham intervention. To our knowledge, this is the first randomized controlled trial (RCT) to apply tDCS with iTBS to facilitate upper-limb motor function in chronic stroke patients. We also expected that the novel DC-iTBS stimulation combined with rehabilitation of the upper extremities would result in greater improvements and have potential to become a routine treatment strategy for stroke patients at hospitals and residential rehabilitation facilities.

## Materials and methods

### Participants

The present study was a single-blinded, RCT pilot study performed at Taipei Medical University (TMU) Hospital (Taipei, Taiwan). Experiments were conducted under a protocol approved by the Joint Institutional Review Board of TMU (registration no. N201702070). All experiments in this research were performed in accordance with relevant guidelines and regulations at TMU. This trial was registered at ClinicalTrials.gov ID no. NCT04369235.

**Fig. 1:**
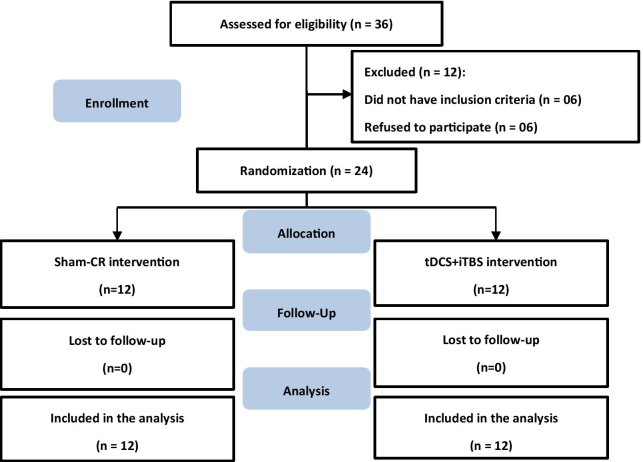
The experimental flowchart of this study. *CR* conventional rehabilitation, *tDCS* transcranial direct current stimulation, *iTBS* intermittent theta burst stimulation

Potential participants were identified between June 2017 and June 2019 from TMU Hospital through convenience sampling. Out of 36 stroke survivors who underwent an initial screening, 24 participants were eligible according to the study inclusion criteria and consented to take part. A flowchart of inclusion of patients is presented in Fig. 1. Participant characteristics are presented in Table 1. Time since the stroke and stroke location were determined from medical records. We included patients with ischemic or hemorrhagic chronic stroke (within 6 months to 5 years after onset) diagnosed by a neurologist and meeting the following inclusion criteria: (1) aged ≥ 20 years; (2) unilateral cerebral stroke with hemiplegia and Brunnstrom stage IV or V; and (3) adequate understanding of verbal/written information and physically able to complete the motor learning of functional tasks with the affected hand. Exclusion criteria were as follows: (1) lower motor neuron impairment, (2) unstable autonomic nervous system, (3) extremely sensitive to electrical stimulation and could not tolerate it, (4) contractures in the upper extremity or limitations of joint motion, (5) severe spasticity, (6) myositis ossificans, (7) a history of arrhythmia, (8) a medical electronic device implant, such as a pacemaker, (9) decubitus or scalp wounds, (10) metal head or neck implants, (11) severe cognitive dysfunction or active psychiatric diseases, such as schizophrenia or dissociative identity disorder, (12) a history of seizures or organic brain disease, (13) severe traumatic brain injury, (14) drug or alcohol abuse, and (15) a malignant tumor or an autoimmune rheumatic disease, such as systemic lupus erythematosus, rheumatoid arthritis, or ankylosing spondylitis.

**Table 1:** Clinical and demographic characteristics of study participants

	Sham NIBS (*N* = 12)	Real NIBS (*N* = 12)	Total (*N* = 24)	*p* value
Age (years)				
Mean ± SD	65.92 ± 13.98	62.08 ± 15.58	64.00 ± 14.61	0.53
Median	67.5	65.5	66.5	
Sex (M/F)	7/5	9/3	17/7	0.67
Stroke etiology (ischemic/hemorrhagic)	8/4	8/4	16/8	1.00
Affected hemisphere (right/left)	7/5	7/5	14/10	1.00
Time since stroke (month)		8	7	
Mean ± SD	18.00 ± 17.42	20.25 ± 14.74	19.13 ± 15.82	0.34
Median	9.5	14	11	
FMA-UE baseline				
Mean ± SD	45.17 ± 17.18	37.50 ± 20.54	41.33 ± 18.93	0.37
Median	51	39	43.50	
JTT (total time, s) baseline				
Mean ± SD	179.54 ± 90.79	209.71 ± 100.27	194.62 ± 94.81	0.62
Median	135.76	195.67	144.30	
FNT baseline				
Mean ± SD	29.25 ± 15.86	20.46 ± 16.55	24.85 ± 16.47	0.20
Median	32.75	23.00	27.25	

*Sham NIBS* sham transcranial direct current stimulation (tDCS) + intermittent theta burst stimulation (iTBS) with conventional rehabilitation, *Real NIB* tDCS + iTBS with conventional rehabilitation, *SD* standard deviation, *M/F* male/female, *FMA-UE* Fugl-Meyer Assessment-Upper Extremity, *JTT* Jebsen–Taylor test, *FNT* Finger-to-Nose test, *p* value = difference between the sham NIBS group and real NIBS group

### Experimental protocol and design

Participants were randomly assigned to receive real NIBS which included conventional rehabilitation (CR) combined with real-tDCS + iTBS output (the real NIBS group) or sham NIBS with sham-tDCS + iTBS output (the sham NIBS group) using a computer-generated randomization scheme. Randomization, functional outcome measurements, and data analyses were performed by trained research staff who were not involved in the intervention. All participants received similar durations of CR therapy in both the real and sham-NIBS groups. CR treatments (including fine motor skill training, normal limb posture, active range-of-motion exercises, and muscle strengthening exercises) were conducted by certified occupational therapists who were blinded to the group assignment and were trained according to the investigator’s protocol. This experimental protocol consisted of 18 sessions of a 1-h CR program (i.e., 3 days a week for 6 weeks), where a 20-min NIBS current was simultaneously applied at the beginning of the 1 h of CR in all sessions.

**Fig. 2:**
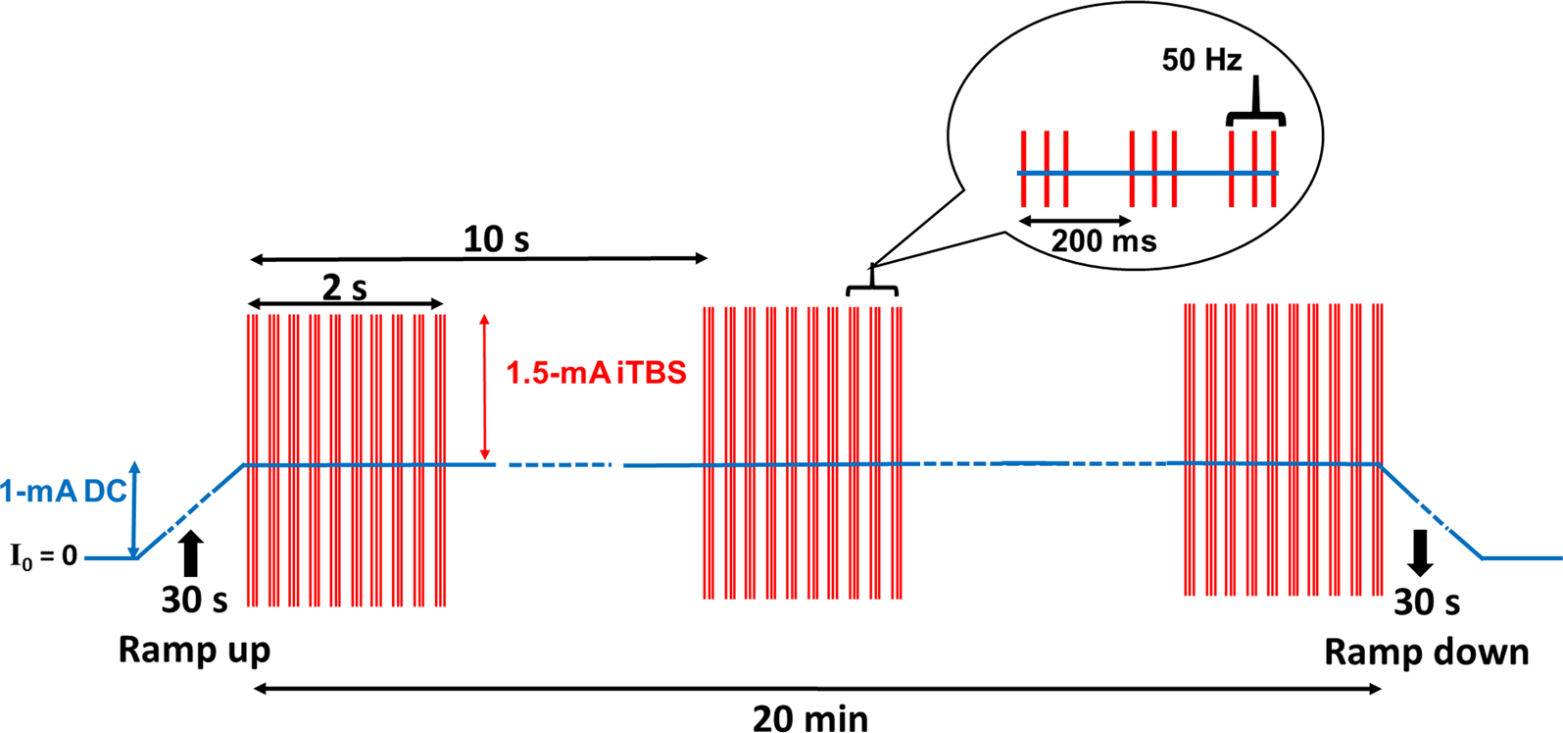
The combined DC-iTBS protocol was iTBS of 1.5-mA intensity superposed on continuous 1-mA DC. The basic pattern of iTBS consisted of bursts containing three biphasic pulses at 50 Hz repeated at 200 ms intervals; and a 2 s train of iTBS was repeated every 10 s. For each stimulation, current intensity was ramped up to 1 mA over 30 s at the beginning, applied for 20 min and then ramped down to 0 mA over 30 s at the end of stimulation

Transcranial current stimulation was utilized from our previously developed novel and portable device [12]. This device can electrically generate a DC combined with iTBS, which was applied using a pair of saline-soaked electrode sponges (with a 7 × 5 cm or 35 cm^2^ surface area), with firm fixation of the electrodes to the scalp during CR treatment. For the combined DC-iTBS protocol, iTBS of 1.5-mA intensity was superposed on continuous 1-mA DC [12], as shown in Fig. 2. The basic pattern of iTBS stimulation consisted of bursts containing three biphasic pulses at 50 Hz repeated at 200 ms intervals. A 2 s train of iTBS was repeated every 10 s [14, 30]. Participants in the real NIBS group received a current intensity of 1 mA (DC) combined 1.5 mA (iTBS) for 20 min, and the anode electrode was placed over the primary motor cortex (M_1_) scalp location (C3 or C4 according to the EEG 10/20 system) of the affected hemisphere, and the cathode was placed on the contralateral back of the shoulder region. For each stimulation, current intensity was ramped up to 1 mA over 30 s at the beginning, applied for 20 min and then ramped down to 0 mA over 30 s at the end of stimulation.

For the sham NIBS group, participants received an intervention as the active tDCS + iTBS group except that the stimulation intensity current was ramped up to 1 mA over 30 s and then the current was ramped down to 0 mA over 30 s throughout the entire process while the electrode sponges were kept on the participants’ scalp for 20 min to mimic real stimulation. All participants were informed that they would likely feel a warm, light itching sensation under the stimulation electrodes at the beginning and these feelings might disappear after a short time.

### Outcome measures

#### Fugl-meyer assessment-upper extremity (FMA-UE)

In the week before treatment initiation (baseline), clinical and demographic characteristics of each patient were collected. For all groups, functional outcomes were measured at the baseline, immediately after, and 6 weeks after 18 therapeutic sessions (post-treatment) by a researcher blinded to which subjects were receiving which treatment. The FMA-UE was performed (score ranges 0 ~ 66) to assess upper-limb motor recovery [31]. This test is considered to be a core measures to detect changes in motor recovery in stroke and rehabilitation trials [32]. Each task is rated from 0 to 2, with a higher score representing better performance [33, 34].

#### Jebsen-taylor hand function test (JTT)

The JTT is widely used to assess a broad range of functional hand motor skills that reflect activities of daily living [35]. JTT was shown to be a reliable and valid tool, for which normative data are available for all ages and in both genders [35, 36]. We included seven subtests of the JTT in our analyses: writing, turning cards, picking up small objects, picking up beans with a teaspoon, stacking checkers, lifting light cans, and lifting heavy cans. Participants were instructed to perform a task as fast and as accurately as possible. The total JTT time and subtest JTT times (maximum measured time was 45 s for each subset) were recorded for analysis.

#### Finger-to-nose test (FNT)

The FNT is often used to assess upper-limb coordination after a stroke [31, 37]. While a patient sits on a chair in front of the examiner, they were asked to alternately touch their nose and then extend their finger to touch the examiner's finger as quickly as possible. The numbers of touch events within 1 min were recorded for analysis.

### Statistical analysis

Statistical analyses were performed using SigmaPlot, vers. 10.0 (Systat Software, CA, USA). Normality was assessed using Kolmogorov–Smirnov tests. Most of the outcome measures were not normally distributed, so nonparametric statistical analyses were used to assess the data. Baseline demographic and clinical measures (mean, standard deviation (SD), and median) were compared between two groups using the Mann–Whitney *U*-test for continuous variables and Pearson’s Chi-squared test for categorical variables.

Functional outcome measures were analyzed by Wilcoxon matched-pairs signed-ranks test to investigate time effects (baseline and post-treatment) within groups. The Mann–Whitney rank sum test was used to test differences in outcomes among groups. Change in subset JTT times after stimulation in percentage were calculated according to the following equation: ${\text{Change in }}\% \, = \,\left( {{\text{Baseline}}/{\text{Post-treatment}} \times \,{\text{1}}00{-}{\text{1}}00} \right)$[38]. Statistically significant differences were considered at *p* < 0.05.

## Results

### Baseline characteristics of subjects

Thirty-six subjects were screened to participate in our study, and 12 subjects were excluded. We included 24 stroke survivors who met the inclusion criteria; 12 were allocated to the real NIBS group and 12 to the sham NIBS group. Baseline characteristics of all patients, including demographics, stroke characteristics, and functional outcomes are summarized in Table 1. The two groups did not significantly differ in demographics (age and sex), stroke condition (time after stroke, Brunnstrom stage, stroke etiology, and affected hemisphere), or functional outcomes (FMA-UE, JTT, and FNT), indicating that randomization ensured baseline comparability between groups. No patient in either group experienced any adverse events during or after the tDCS + iTBS treatment.

### Functional tests results

Table 2 shows summary information for the outcomes of all functional tests. For the FMA-UE, Wilcoxon matched-pairs signed-ranks test revealed a significant time effect with an increase in mean FMA-UE scores in both the sham NIBS group (from 45.17 ± 17.18 at the baseline to 52.17 ± 15.03 post-stimulation; *p* < 0.001) and the real NIBS group (from 37.50 ± 20.54 at the baseline to 48.33 ± 16.55 post-stimulation; *p* < 0.001). However, in our initial analysis of comparing differences in FMA-UE score between the real and sham NIBS groups, we found no significant differences (*p* = 0.104). Contrarily, the signed-ranks test indicated that total JTT times (from 209.71 ± 100.27 s at the baseline to 177.94 ± 97.64 s post-stimulation; *p* < 0. 01) and the number of FNT touch events (20.46 ± 16.55 at the baseline to 33.75 ± 13.73 post-stimulation; *p* < 0. 05) significantly improved only in the real NIBS group.

**Table 2:** Functional tests results (FMA-UE, JTT, and FNT) with significance values

	Sham NIBS (*N* = 12)		Real NIBS (*N* = 12)		*p* value
	Mean ± SD	Median	Mean ± SD	Median	
FMA-UE					
Baseline	45.17 ± 17.18	51	37.50 ± 20.54	39	
Post-treatment	52.17 ± 15.03***	60	48.33 ± 16.55***	51	
Δpre-post	7.00 ± 7.35	3.5	11.33 ± 10.52	9	0.104
JTT (total time, sec)					
Baseline	179.54 ± 90.79	135.76	209.71 ± 100.27	195.67	
Post-treatment	174.43 ± 94.07	115.64	177.94 ± 97.64**	133.49	
Δpre-post	5.11 ± 13.26	4.06	31.76 ± 49.78	18.52	0.016
Finger to nose					
Baseline	29.25 ± 15.86	32.75	20.46 ± 16.55	23.00	
Post-treatment	32.04 ± 17.41	35.25	33.75 ± 13.73*	34.75	
Δpre-post	2.79 ± 5.17	0	13.29 ± 18.60	7	0.037

*Sham NIBS* sham transcranial direct current stimulation (tDCS) + intermittent theta burst stimulation (iTBS) with conventional rehabilitation, *Real NIBS* tDCS + iTBS with conventional rehabilitation, *SD* standard deviation, *M/F* male/female, *FMA-UE* Fugl-Meyer Assessment-Upper Extremity, *JTT* Jebsen-Taylor test,*FNT* Finger-to-Nose test, *p* value = independent sample *t*-test for difference between mean changes from baseline of the sham NIBS group versus the real NIBS group. *p < 0.05 or **p < 0.01 or ***p < 0.001 paired *t*-test of within-group differences of baseline—post-treatment

For further statistical analysis, the real NIBS substantially led to a significant reduction in total JTT times relative to the baseline (i.e. Δ pre-post) compared to that of control values (31.76 ± 49.78 and 5.11 ± 13.26 s, respectively; *p* = 0. 016). Similarly, a significant increase was noted in the number of FNT touch events when the value in the real group was compared to that in sham group (13.29 ± 18.60 vs. 2.79 ± 5.17, respectively; *p* = 0. 037).

### Jebsen-Taylor hand function test (JTT)

For the analyses of seven subtests of the JTT, there were no detectable differential time effects within groups (Table 3). However, compared to the sham group, the real NIBS group exhibited a significantly decreased change in the JTT for lifting light cans (-19.22% ± 25.70% and -2.10% ± 10.73%, respectively; *p* = 0. 034) and lifting heavy cans (-20.09% ± 26.02% and 0.64% ± 15.42%, respectively; *p* = 0. 027). The negative percentage change indicates a reduction in time of JTT subtests and consequent improved hand function. These results indicated that the tests requiring more proximal arm motions (proximal tasks including moving light and heavy cans) tended to improve more than those that required finer motor control (writing, turning cards, picking up small objects, picking up beans with a teaspoon, and stacking checkers) in the NIBS group.

**Table 3:** Effects of non-invasive brain stimulation (NIBS) with transcranial direct current stimulation (tDCS) + intermittent theta burst stimulation (iTBS) waveform on subtests of the Jebsen-Taylor test (JTT)

	Sham NIBS (*N* = 12)			Real NIBS (*N* = 12)			*p* value
	Baseline (Mean ± SD)	Post (Mean ± SD)	Change in % (Mean ± SD)	Baseline (Mean ± SD)	Post (Mean ± SD)	Change in % (Mean ± SD)	
Writing	40.10 ± 9.01	38.61 ± 9.50	− 3.52 ± 9.82	41.31 ± 7.17	37.15 ± 9.14	− 9.40 ± 17.37	0.362
Turning cards	20.81 ± 15.35	19.59 ± 15.68	− 6.62 ± 12.87	28.11 ± 17.74	24.99 ± 16.53	− 8.13 ± 26.81	0.907
Picking up small objects	28.51 ± 12.31	25.96 ± 14.25	− 10.48 ± 15.73	31.34 ± 14.62	28.01 ± 15.26	− 10.27 ± 20.50	0.814
Picking up beans	29.17 ± 14.62	27.29 ± 14.38	− 3.84 ± 21.21	32.40 ± 13.13	27.33 ± 12.91	− 14.28 ± 21.00	0.238
Stacking checkers	22.37 ± 17.33	24.56 ± 18.22	18.79 ± 54.02	25.89 ± 20.04	22.51 ± 18.27	− 1.44 ± 37.14	0.254
Lifting light cans	20.21 ± 15.86	19.75 ± 15.92	− 2.10 ± 10.73	25.01 ± 17.81	18.64 ± 14.96*	− 19.22 ± 25.70	0.034
Lifting heavy cans	18.73 ± 16.03	18.66 ± 16.06	0.64 ± 15.42	25.64 ± 17.54	19.32 ± 16.04*	− 20.09 ± 26.02	0.027

*Sham NIBS* sham tDCS + iTBS with conventional rehabilitation, *Real NIBS* tDCS + iTBS with conventional rehabilitation, *SD* standard deviation Change in % = (Baseline/Post × 100 – 100); *p* value = independent-sample *t*-test for difference between mean change in % from the baseline of the Sham-CR group versus tDCS + iTBS group. *p < 0.05 paired *t*-test for within-group differences in baseline—post treatment. Negative values indicate a reduction in time of JTT subtests and consequent performance improvement

## Discussion

The present study demonstrates that the tDCS + iTBS combined with CR had a significant additive effect on motor function, as reflected in JTT and FNT results, compared to that found in the sham group, as shown in Table. 2. In addition, improvements in the JTT tests requiring more proximal arm motions (lifting light/heavy cans) were more significant in the real NIBS group compared to values in the sham group. These results suggest that tDCS-iTBS combined with a conventional rehabilitation intervention may be a promising strategy to restore motor function in chronic hemiplegic patients.

Few patients (2 of 24 subjects) in this study experienced mild nausea or a headache during treatment, which was relieved after the end of treatment. Generally, they usually adapted within four times, and the symptoms completely disappeared. No other adverse reactions were noted in this study. The safety of repetitive sessions of iTBS 1200 over ipsilesional M_1_ of subacute stroke patients has long been confirmed [39]. iTBS of the affected motor cortex during the acute phase in patients with hemiparesis due to capsular infarction also appeared to enhance motor recovery without side effects [40].

New studied forms of stimulation, such as tDCS and iTBS, may have improved neuromodulatory efficacy due to increased localization [41, 42] or may be more effective because of physiological effects [14, 43–45]. In our study, motor function of the upper limb was measured using the FMA-UE scale. It changed significantly after tDCS combined with iTBS stimulation from 37.50 ± 20.54 to 48.33 ± 16.55. Our results are consistent with data from a recent study that reported a significant change in FMA-UE scores in subacute stroke survivors using tDCS combined with functional electrical stimulation. They further reported that the mean difference in FMA-UE scores between the baseline day 0 and day 21 indicated significant improvement (Δ pre-post = 6.20 ± 0.92, *p* < 0.05) [46]. However, in our present study, the mean difference in FMA-UE in the real group indeed showed a dramatic improvement (Δ pre-post = 11.33 ± 10.52, *p* = 0.104, Table 2), but the difference did not reach statistical significance. This discrepancy may have resulted from some differences in the experimental protocols between the two clinical trials such as stroke etiology, time since the stroke, and stimulation intensity etc.

With other outcome measures in our study, such as JTT scores, we only detected a significant improvement in the real NIBS group that received the tDCS + iTBS with CR intervention (Δ pre-post = 31.76 ± 49.78). Contrarily, the sham group (which only received a CR intervention) showed an insignificant change in JJT scores (Δ pre-post = 5.11 ± 13.26). A similar trend was also observed for FTN scores with significant improvement in the real NIBS intervention compared to scores of the sham intervention. Thus, our study demonstrated that the special current waveform of the tDCS combined with iTBS may facilitate upper-limb motor function in chronic stroke patients.

The combined stimulation with anodal DC-iTBS induces a better LTP- like neuroplasticity, as evidenced by detecting a significant improvement in MEP data in an animal study [12]. As the previous literature is deficit in explaining the effects of combined tDCS-iTBS stimulation in humans. The possible mechanisms for LTP-like neuroplasticity may be due to the coupling of theta and gamma rhythms which influences intrinsic circuitry of the motor cortex [14], resulting in increased amplitude of later I-waves with iTBS protocol [43, 47]. While tDCS acts through membrane polarization and NMDA receptor-mediated glutamatergic synaptic transmission [48, 49]. Therefore, iTBS-like anodal DC stimulation simultaneously induced the two abovementioned neuroplastic mechanisms of iTBS and tDCS which lead to a summation effect on the neuroplasticity. When compared to traditional anodal tDCS, the DC-iTBS-like technique may have a quicker and more intense impact.

According to Bienenstock-Cooper Munroe (BCM) theory [50], the synaptic plasticity may rely on the stimulation frequency. LTD was induced by tetanic stimulation over a range of low frequencies (generally ≤ 1 Hz), whereas LTP was induced by stimulation at a range of high frequencies [50]. These findings again support our speculation that the frequency rhythm of iTBS may play an important role on the LTP modulation. The BCM theory further indicated that the existence of the intensity-dependent plasticity, i.e. a synaptic depression at the activated synapses if the postsynaptic cell is not sufficiently depolarized. Although our present study did not determine the effects of waveform intensity on clinic outcomes, we speculate that magnitude of stimulation intensity may have bidirectional effects on synaptic plasticity, i.e. LTD and LTP. Nevertheless, the neuromodulatory mechanisms in synaptic plasticity in human warrant further exploration.

Although the exact neural mechanisms underpinning the effects of the different approaches remain unclear, a recent functional magnetic resonance imaging (fMRI) study suggested that better attentional processing after iTBS could be due to increased functional connectivity in this network [51]. iTBS tends to mimic endogenous theta rhythms in the brain's physiology and induces longer-lasting effects of LTP-like neuronal plasticity [52]. Our previous animal study demonstrated that LTP-like plasticity can be dramatically induced in rats using combined tDCS and iTBS protocols. In the present study, the real NIBS utilizing combined tDCS and iTBS protocols significantly improved upper-extremity motor functions in chronic stroke patients. These results imply that our novel coupling protocol may upregulate ipsilesional cortical excitability in clinical hemiplegic patients. Transcranial electrical stimulation may simultaneously foster other rehabilitation benefits, such as decreased muscle spasticity [53], increased muscle power [54], and other possible physiological responses [53]. Thus, in future studies, these accessory clinical benefits of transcranial neuromodulation warrant further exploration.

The tDCS device typically includes an electrode stimulating anode and a cathode with an adjustable DC stimulator. This transcranial stimulation technology is widely used as a safe form of NIBS. A systematic article examined over 33,000 sessions and 1000 patients who had repeatedly received tDCS and found no evidence indicating that the NIBS protocols caused serious brain injury in human trials [7]. A low-intensity current of 0.5 ~ 2.0 mA is the conventional stimulation intensity, whereas the safety and tolerability of 4-mA stimulation were also tested in clinical trials by placing two electrode sponges on the scalp to stimulate the target cortical region [55, 56]. In this study, we used a pair of 7 × 5-cm^2^ electrode sponges to implement the combined DC-iTBS protocol, where iTBS of 1.5-mA intensity was superposed on a continuous 1-mA DC current. Liebetanz et al. stated that brain lesions occurred at a threshold current density of 142.9 A/m^2^ [57]. Our maximal current density was ~ 7.1 A/m^2^, which should be far from the safe and tolerable threshold for transcranial neuromodulatory interventions. Thus, this clinical trial demonstrated the feasibility and safety of the combined DC-iTBS protocol as evidenced by no severe adverse events among all recruited participants.

tDCS modulates the potential of the resting membrane and synaptic activity to improve or depress excitability based on the polarity and duration of stimulation. For a short stimulation duration, neurons near the anode are hypopolarized, while those proximal to the cathode are hyperpolarized. tDCS alters membrane potentials through calcium and sodium channels and other processes such as γ-aminobutyric acid (GABA) inhibition [58–60]. For a long period of stimulation, anodal (a)-tDCS promotes synaptic activity parallel to long-term potentiation and cathodal (c)-tDCS replicates long-term depression [49]. Long-lasting effects occur through N-methyl-D-aspartate receptors and neurotrophic factors such as brain-derived neurotrophic factor [61, 62].

Our present study used a-tDCS which is supported by another study using a- and c-tDCS, and they reported that both can improve neglected symptoms in stroke patients [63]. This was evidenced by the previous literature which used inhibitory (i.e., low-frequency) or facilitatory (i.e., high-frequency) repetitive TMS to impact posterior parietal cortex (PPC) function in humans [64] and c-tDCS in cats [65, 66]. If the right hemisphere is damaged, homologous regions of the left hemisphere that usually receive inhibitory projections from the opposite hemisphere become dysregulated. This probably causes an unopposed orienting reaction to the right side. If dysregulation causes inhibition on the intact side, attentional bias occurs towards the ipsilesional side of space [67] suggesting corresponding interhemispheric inhibition by both parietal lobes.

**Limitations:** There are several limitations to the current study. First, we did not include conventional tDCS or iTBS stimulation as a comparison group in our study. Although our previous animal study has demonstrated that the combined DC-iTBS waveform induced a better neuroplastic effect compared to that in the conventional tDCS alone [12], we still cannot assert that the therapeutic effect of the real NIBS group completely resulted from the coupling effect of these two approaches in the clinic trial. Thus, it needs further exploration for this possibility.

Second, the study was single-blinded due to characteristics of the tDCS device. This could potentially have caused bias when conducting the sessions. Third, the sample size was small, so applications in large population are required in the future. Fourth, this study tested electrostimulation only on chronic stroke patients with upper-limb impairment (Brunnstrom stage IV or V); other stroke categories with lower-limb impairments or subacute or chronic conditions need to be tested using this kind of electrostimulation. Fifth, other neurological conditions, like Alzheimer’s and Parkinson’s disease, will be tested in the future for the effect of electrostimulation on the brain for improvements in cognition, memory, and functional activity status to develop smart electrostimulation devices for use in clinical rehabilitation.

## Conclusions

In conclusion, we provide evidence that patients who underwent combined ipsilesional DC-iTBS with conventional rehabilitation had greater impacts than patients who underwent sham stimulation-conventional rehabilitation in statistically significant clinical responses of total JTT time and FNT after a stroke. The preliminary results in upper-limb functional recovery may have potential therapeutic benefits, and large-scale studies are needed to confirm and validate our current results.

## Data Availability

Not applicable.

## Abbreviations

CR: Conventional rehabilitation
cTBS: Continuous theta burst stimulation
DLPFC: Dorsolateral prefrontal cortex
FMA-UE: Fugl-Meyer Assessment Upper Extremity
FNT: Finger-to-Nose test
GABA: γ-Aminobutyric acid
HD-tDCS: High definition transcranial direct current stimulation
iTBS: Intermittent theta burst stimulation
JTT: Jebsen-Taylor hand function test
LTD: Long-term depression
LTP: Long-term potentiation
MEP: Motor evoked potential
NIBS: Non-invasive brain stimulation
PPC: Posterior parietal cortex
ProcTBS: Prolonged continuous
RCT: Randomized controlled trial
rTMS: Repetitive transcranial magnetic stimulation
SMD: Standardized mean deviation
TBS: Theta burst stimulation
tDCS: Transcranial direct current stimulation
